# Effectiveness of a Community-Based Organization—Private Clinic Service Model in Promoting Human Papillomavirus Vaccination among Chinese Men Who Have Sex with Men

**DOI:** 10.3390/vaccines9111218

**Published:** 2021-10-20

**Authors:** Zixin Wang, Yuan Fang, Paul Shing-fong Chan, Andrew Chidgey, Francois Fong, Mary Ip, Joseph T. F. Lau

**Affiliations:** 1 JC School of Public Health and Primary Care, Faculty of Medicine, The Chinese University of Hong Kong, Hong Kong, China; pchan@link.cuhk.edu.hk (P.S.-f.C.); mitk@cuhk.edu.hk (M.I.); 2 Department of Early Childhood Education, The Education University of Hong Kong, Hong Kong, China; lunajoef@gmail.com; 3 AIDS Concern, Hong Kong, China; andrew.childgey@aidsconcern.org.hk; 4 Neohealth, Hong Kong, China; drfong@neohealth.com.hk

**Keywords:** HPV vaccination, men who have sex with men, online health promotion, outcome and process evaluation

## Abstract

This study evaluated the effectiveness of the community-based organization (CBO)-private clinic service model in increasing human papillomavirus (HPV) vaccination uptake among unvaccinated men who have sex with men (MSM) in Hong Kong during a 12-month follow-up period. A CBO-private clinic model was implemented to promote HPV vaccination among Chinese MSM. A CBO with good access to MSM approached MSM aged 18–45 years who had never received an HPV vaccination, invited them to receive an online health promotion, and referred them to receive HPV vaccination at gay-friendly private clinics. A baseline survey and a follow-up evaluation at Month 12 were conducted. A total of 350 participants completed the baseline survey. Among 274 participants who were followed up at Month 12, 46 (16.8%) had taken up at least one dose of HPV vaccination. After adjusting for significant baseline characteristics, the perceived susceptibility (AOR:1.25, *p* = 0.002) and perceived severity (AOR:1.21, *p* = 0.003) of HPV and HPV-related diseases, perceived benefits (AOR:1.16, *p* = 0.03), self-efficacy to receive HPV vaccination (AOR:1.37, *p* = 0.001), and behavioral intention to take up HPV vaccination at baseline (AOR:6.99, *p* < 0.001) significantly predicted HPV vaccination uptake. The process evaluation of the program was positive. The CBO-private clinic service model was helpful in increasing HPV vaccination uptake among MSM.

## 1. Introduction

Across countries, men who have sex with men (MSM) have a much higher risk of contracting human papillomavirus (HPV) and its related diseases (e.g., genital warts and penile cancers) than the general male population [1,2,3,4]. As reported by a meta-analysis, the overall prevalence of genital HPV infection was very high among both human immunodeficiency virus (HIV)-negative (63.9%) and HIV-infected MSM (92.6%) [5]. Moreover, MSM’s risk of genital warts and anal cancers were much higher than the general population [6,7]. The prevalence of genital warts was 13.2–58.6% among MSM [6,7]. The overall prevalence of high-grade anal histological lesions was 23.9% among HIV-infected MSM and 15.2% among HIV-negative MSM [5]. The HPV-related cancer risk was the highest among HIV-infected MSM, which accounted for 9.9% of the MSM population in China in 2016 [8]. Slow clearance and increased persistence of high-risk HPV might explain why HIV-infected MSM are more susceptible to HPV and its related diseases compared with HIV-negative MSM [9,10,11,12,13]. A previous study showed that men with HIV infection had lower high-risk HPV clearance [9]. In-vitro studies showed increased expression of HPV E1 and L1 viral gene in the presence in HIV trans-activator of transcription (tat) protein [10]. HIV-tat protein was shown to enhance HPV transcription during HPV replication [11]. Moreover, uncontrolled HIV infection might increase the persistence of high-risk HPV through altered cell-mediated immunity, local molecular interactions, and reduced tight junction function [12,13]. In Hong Kong, among MSM with an experience of HPV screening, 25% were diagnosed with HPV infection [14].

HPV vaccination is highly effective in preventing vaccine-type genital warts and cancers among MSM [15,16] and other HPV-related diseases [17]. HPV vaccination provides maximum benefit if a person receives it before he/she is sexually active, and its efficacy is lower for MSM aged up to 45 years who have an HPV infection [18,19]. However, the Advisory Committee on Immunization Practices of the United States Centers for Disease Control and Prevention (CDC) recommends that people having an HPV infection should still receive HPV vaccination if they are in the appropriate age group because vaccination may protect them against high-risk HPV types that they have not yet acquired [20]. 

The CDC recommends MSM aged ≤45 years to receive the HPV vaccination [21,22]. In the United Kingdom, national programs provide free HPV vaccination for MSM aged up to 45 years attending sexual health and HIV clinics [23,24]. In 2017, the Victorian Government of Australia rolled out a free time-limited HPV vaccination catch-up program for MSM aged up to 26 years [25]. Relatively high HPV vaccination uptake was observed among MSM in national programs in developed countries, ranging from 37.6% in the United States [26], 42.6% in Australia [25], 49.1% in England [27], to 63.7% in Scotland [24]. However, in Hong Kong, there is no free or subsidized HPV vaccination program for MSM or other male populations. HPV vaccination uptake among the male population is very low in Hong Kong [28,29,30].

A randomized controlled trial (RCT) was conducted between 2017 and 2019 among MSM in Hong Kong to evaluate online intervention promoting HPV vaccination [31]. The results showed that watching a 5-min online video based on the Health Belief Model [32] and receiving brief motivational interviewing (MI) through telephone was effective in improving HPV vaccination uptake over a 24-month study period [31].

Community-based organization (CBO) in Hong Kong has a good position of promoting HPV vaccination among local MSM. First, half of MSM usually received HIV prevention services provided by local CBO (e.g., 45.5–51.5% of the HIV testing and counseling services) [29,33]. Second, it is easier to promote HPV vaccination by CBO staff, as it is naturally for MSM to receive HIV/sexually transmitted infections (STI)-related information from them. Participants may feel less stigmatized and embarrassed. Third, we found that a cue to action, a construct of the HBM [32], was strongly associated with the acceptability of HPV vaccination among local MSM. 

Suggestion from peer CBO workers can serve as a strong cue to action [29]. Moreover, these CBOs have been working closely with gay-friendly private clinics providing STI screening and treatment for MSM. A CBO having good access to local MSM adapted the online intervention developed by the aforementioned RCT and initiated a CBO-private clinic service model promoting HPV vaccination for MSM in August 2019. The CBO approached MSM using its network, provided online intervention promoting HPV vaccination, and referred interested participants to gay-friendly private clinics in its service network for vaccination uptake. This study evaluated the effectiveness of the CBO-private clinics service model in increasing HPV vaccination uptake among unvaccinated MSM in Hong Kong during a 12-month follow-up period. Baseline factors predicting HPV vaccination uptake were investigated and process evaluation was performed.

## 2. Materials and Methods

### 2.1. Study Design

**Figure 1:**
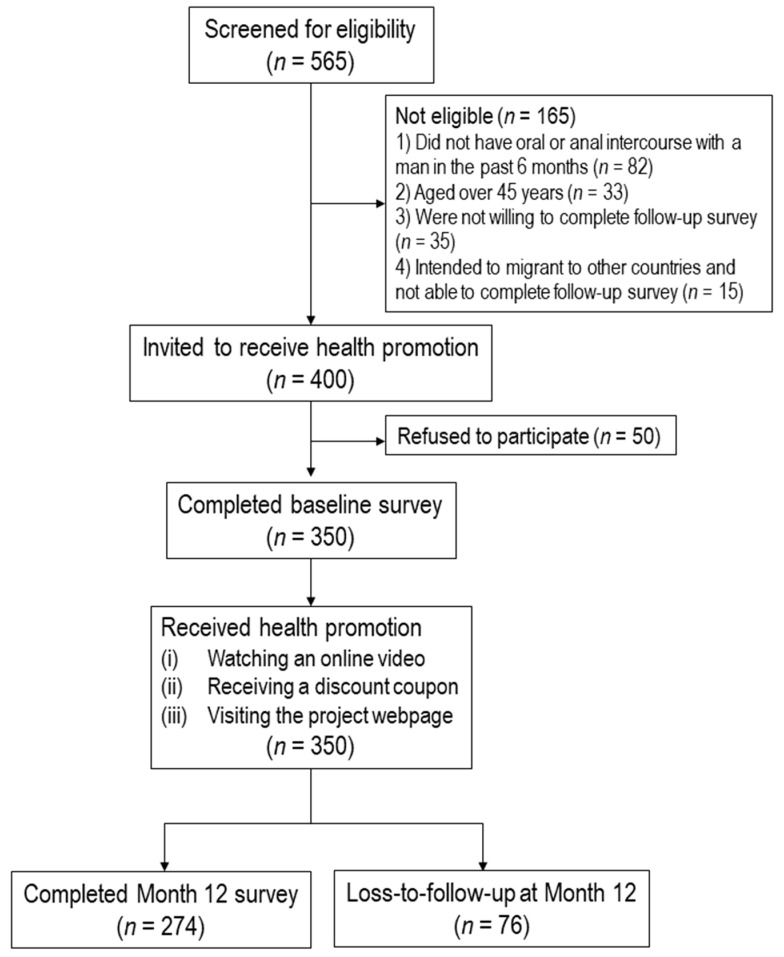
Flowchart diagram.

This longitudinal study was conducted from August 2019 to April 2021. All participants completed a telephone survey before they received online interventions promoting HPV vaccination, and completed another telephone survey 12 months after the baseline survey. There was no control or comparison group. The flowchart diagram was shown in Figure 1. The study was registered at ClinicalTrial.gov (NCT04815837).

### 2.2. Participants

Inclusion criteria were: (1) Hong Kong Chinese speaking males aged 18–45 years old, (2) self-reported having had oral or anal intercourse with at least one man in the last six months, (3) never received HPV vaccination, and (4) willing to complete a follow-up telephone survey 12 months after the baseline survey.

### 2.3. Sampling and Data Collection

Trained staff of a CBO providing HIV-related services recruited participants through multiple sources, including outreaching in gay bars or saunas in Hong Kong, online recruitment, and referrals made by peers and other CBOs. Detailed recruitment procedures were reported in a published paper [14]. Interested participants contacted fieldworkers through WhatsApp, telephone, email, or other means. Participants were guaranteed anonymity during the study, and had the right to end participation in the study at any time. Their refusal or withdrawal from the study would not affect their access to any future services. Verbal instead of written informed consent was obtained due to maintaining anonymity, and the fieldworkers signed a form pledging that the participants had been fully informed about the study. Multiple forms of contact information were obtained to make appointments for conducting the baseline and Month 12 telephone interview. A HK$25 (approximately US$3.2) supermarket or café coupon was mailed to participants as compensation of their time after they completed each survey. Ethics approval was obtained from the Survey and Behavioral Research Ethics Committee of the Chinese University of Hong Kong (reference number: KPF18HLF22).

### 2.4. Baseline Telephone Survey and Health Promotion

By appointment, the CBO staff conducted the 15-min baseline survey by telephone. All participants received the following health promotion components:

(1) Watching an online video promoting HPV vaccination for MSM: The online video developed by our team in a previous RCT was modified for this study [31]. The modifications included: (i) cost of HPV vaccination was updated based on the recent market rate, and (ii) information of collaborative clinics (e.g., location and contacts) were added. The video was guided by the HBM [32]. 

In the video, a peer MSM talked about the risk of having penile or anal cancers among MSM, the severe consequences of HPV-related diseases, high efficacy, and long protection duration of HPV vaccination. The video also contained flashes of scary images of genital warts and penile and anal cancers to increase the perceived severity. The peer MSM also emphasized that HPV vaccination was a worthy long-term investment for their health. He demonstrated the procedures for receiving HPV vaccination in one of the collaborative private clinics, which was convenient, caring, privacy guaranteed, and non-judgmental. Participants could not fast-forward or skip any part of the video.

(2) Each participant was sent a discount coupon through SMS/WhatsApp and could enjoy a 10% discount for taking up three doses of HPV vaccines at a collaborative private clinic.

(3) Participants could access the project webpage by scanning the Quick Response (QR) code on the discount coupon. Through the webpage, participants could: (i) watch the aforementioned online video; (ii) read the description of the project, knowledge, and benefits of HPV vaccination, information about HPV/HPV-related diseases that increased the perceived susceptibility and perceived severity; (iii) access a discussion forum containing positive feedbacks of peers who have taken up HPV vaccination. These testimonials provided cue to action supporting HPV vaccination; and (iv) obtain the contact information of the project staff and information about the collaborative private clinics.

(4) Five reminders were sent to participants by SMS/WhatsApp at Month 1, 2, 4, 6, and 8.

### 2.5. Facilitating HPV Vaccination Uptake in Collaborative Private Clinics

During the project period, two types of HPV vaccines were available for males (Gardasil 4-valent and 9-valent vaccines). For participants who decided to take the vaccine in the collaborative private clinics, they could sign up on the project webpage by filling up their contacts and preferable timeslots. The CBO staff would contact them by telephone or WhatsApp to facilitate appointment making to take up HPV vaccination at collaborative private clinics at a 10% discount from the market rate (about HK$4200 or US$542 for three doses). Participants could also directly contact the collaborative clinics to make an appointment. The project did not limit participants’ choice for taking up HPV vaccination in other places.

### 2.6. Month 12 Follow-up Telephone Survey

Participants were invited to complete a follow-up telephone survey 12 months after the baseline survey. Up to five calls were made at different timeslots during weekdays and/or weekends before considering a participant as a dropout.

### 2.7. Measurements

#### 2.7.1. Primary Outcome

The primary outcome of the study was the validated uptake of any doses of HPV vaccination within a 12-month follow-up period. To validate HPV vaccination uptake, the participants were requested to send the project staff an image of their receipt without personal identification after they received each dose of HPV vaccines. The same approach to validate HPV vaccination uptake was used in a previous study [31]. Vaccinated participants were asked about the location and cost of HPV vaccination uptake, whether they experienced side-effects after vaccination, and the severity of those side-effects.

#### 2.7.2. Baseline Characteristics

Various baseline information was collected, such as socio-demographics, sexual orientation, HIV or STI prevention service utilization, history of HIV and other STIs, and queried sexual behaviors, which included anal intercourse with male regular and non-regular sex partners and male sex workers, condomless anal intercourse (CAI) with men, multiple male sex partnerships, sexualized drug use (SDU), and so on. Regular male sex partners (RP) were defined as lovers and/or stable boyfriends, while non-regular male sex partners (NRP) were defined as casual sex partners. In this study, SDU was defined as the use of any psychoactive substance before or during sexual intercourse [34,35].

Six items measured knowledge related to HPV and HPV vaccination. A composite variable was constructed by counting the number of correct responses (ranged from 0 to 6). Five scales assessed perceptions based on the HBM, including a three-item Perceived Susceptibility Scale, four-item Perceived Severity Scale, four-item Perceived Benefit Scale, five-item Perceived Barrier Scale, two-item Cue to Action Scale, and two-item Perceived Self-Efficacy Scale. These scales were modified from those used in a previous study among MSM in Hong Kong [31].

#### 2.7.3. Process Evaluation

At Month 12, participants were asked to evaluate the health promotion (e.g., whether the contents were clear and attractive). Participants who have taken up HPV vaccination were asked additional questions rating their satisfaction of the services provided by the collaborating clinics.

### 2.8. Statistical Analysis

The difference in baseline characteristics between participants who completed Month 12 follow-up and dropouts were compared by using chi-square tests (for categorical variables) or Mann–Whitney U tests (for continuous variables). The subsequent analysis was performed with data from those who had completed both surveys. We used the validated uptake of any doses of HPV vaccination during the follow-up period as the dependent variable and baseline background characteristics (e.g., socio-demographics, sexual orientation, and history of HIV and other STIs) as independent variables. 

Crude odds ratios (OR) predicting that the dependent variable were obtained using logistic regression models. After adjustment for those variables with *p* < 0.05 in the univariate analysis, the association between knowledge and perceptions related to HPV and HPV vaccination and the dependent variable were then assessed by adjusted odds ratios (AOR). Each AOR was obtained by fitting a single logistic regression model, which involved one of the perceptions and the significant background variables. SPSS version 21.0 (Chicago, IL, USA) was used for data analysis, and *p* < 0.05 (two-sided) is considered as statistically significant.

## 3. Results

### 3.1. Baseline Characteristics

Four hundred of the 565 prospective participants were screened to be eligible, 50 of whom refused to participate in the study for time or other logistic reasons, and 350 (87.5%) completed the baseline survey and received health promotion. At Month 12, 274 (78.3%) completed the follow-up survey.

At baseline, about half of the participants were 18–30 years old (50.3%), and with a monthly personal income at least HK$20,000 (US$2565) (57.4%). The majority of the participants were currently single (75.7%), had attained tertiary education or above (86.6%), were employed full-time (82.9%), and identified themselves as homosexuals (91.1%). Among the participants, 1.7%, 8.9%, and 16.9% reported a history of HIV, HPV infection/genital warts, and other STI infection, respectively. In the past six months, 50.3% and 54.6% reported CAI with men and multiple male sex partnerships. 

Regarding knowledge related to HPV or HPV vaccination, 48.9–95.7% gave correct responses to different items. The Cronbach alpha for the five scales based on the HBM were acceptable (0.61–0.85). Single factors for these scales by exploratory factor analysis, which explained 56.2–82.5% of the total variance. Apart from self-reported HIV sero-status (*p* = 0.02), anal intercourse with NRP (*p* = 0.02), and male sex workers (*p* = 0.01), no significant difference was found between participants who completed the Month 12 follow-up and dropouts (Table 1).

### 3.2. HPV Vaccination Uptake

At Month 12, 46 participants self-reported having had taken up at least one dose of HPV vaccination (three doses: *n* = 13; two doses: *n* = 24; and one dose: *n* = 9). All of them were able to provide receipts for verification. The location for receiving HPV vaccination included the collaborative private clinics (28/46, 60.9%) and other private hospitals/clinics (18/46, 39.1%). The participants self-paid HK$2000–6800 (US$ 258–877; median: HK$4200 or US$542) to receive the vaccination. Most vaccinated participants reported no side effects (37/46, 80.4%). The reported side effects included pain at the injection site (5/46, 10.9%), fatigue (4/46, 8.7%), and dizziness (1/46, 2.2%). Most of these side effects were very mild or mild (8/9, 88.9%).

### 3.3. Factors Predicting HPV Vaccination Uptake

Among 274 participants who had completed both baseline and Month 12 surveys, a history of HPV infection and/or genital warts (OR: 6.02, 95%CI: 2.60, 13.94, *p* < 0.001) and utilization of HIV/STI prevention services other than HIV testing at baseline (OR: 1.98, 95%CI: 1.01, 3.89, *p* = 0.046) were associated with higher HPV vaccination uptake during the follow-up period (Table 2).

After adjusting for these significant baseline characteristics, four constructs of the HBM were associated with the dependent variable in expected directions. They were: (1) perceived higher risk of contracting HPV and HPV-related diseases (perceived susceptibility) (AOR: 1.25, 95% CI: 1.09, 1.43, *p* = 0.002), (2) perceived consequences of HPV-related diseases to be severer (AOR: 1.21, 95%CI: 1.07, 1.37, *p* = 0.003), (3) perceived benefits of HPV vaccination (AOR: 1.16, 95% CI: 1.01, 1.33, *p* = 0.03), and (4) perceived self-efficacy of taking up HPV vaccination (AOR: 1.37, 95%CI: 1.14, 1.65, *p* = 0.001). In addition, behavioral intention to take up HPV vaccination at baseline was also associated with higher uptake during the follow-up period (AOR: 6.99, 95% CI: 3.34, 14.60, *p* < 0.001) (Table 3).

### 3.4. Reasons for Not Taking up HPV Vaccination

At Month 12, 228 unvaccinated participants were asked about their reasons for not taking up HPV vaccination. The most commonly mentioned reason was the high cost of HPV vaccination (128/228, 56.1%), followed by feeling unnecessary to receive such vaccination (79/228, 34.6%), concerns about COVID-19 transmission (73/228, 32.0%), and no time to do so (60/228, 26.3%).

### 3.5. Process Evaluation

Among 274 participants who completed the Month 12 survey, 72.3% and 38.7% believed the health promotion video was clear and attractive respectively. The participants thought the video was helpful in the following aspects, such as increasing awareness of benefits of HPV vaccination (73.4%), reducing barriers to receive HPV vaccination (46.0%), and increasing self-efficacy (56.2%) and intention (40.5%) to receive HPV vaccination.

Among 46 vaccinated participants, 50–95.7% rated “satisfactory” to the following services/features of the collaborating clinics: (1) convenience of the location of clinic (89.1%), (2) convenience of the arranged time slot for vaccination (95.7%), (3) waiting time between making appointment and receiving the first dose (84.8%), (4) level of privacy (69.6%), (5) staff’s acceptance of MSM’s subculture (50.0%), (6) explanation made by the staff of private clinics (73.9%), and (7) professionalism of the staff of private clinics (76.1%) (Table 4).

## 4. Discussion

It is important to transform research findings into regular services. We involved CBO and private clinics as key stakeholders at the planning stage. The implementation was smooth and concurred with routine services of CBO and private clinics. The evaluation results showed that the CBO-private clinic service model was helpful in increasing HPV vaccination uptake among MSM in Hong Kong, as 16.8% of participants being followed up at Month 12 had taken up at least one dose of HPV vaccination. We expected most of these participants would complete all three required doses in near future, as all of them settled the payment for the entire package when receiving the first dose and made appointments for receiving the remaining doses. 

Although there was an increase in the cost of HPV vaccines (from HK$3800 or US$490 to HK$4200 or US$542) and there was no MI, the uptake rate was comparable to the intervention group (17.3%) in previous RCT [31]. However, the HPV vaccination uptake in this study was lower than those in the United States (19.4–45%) [36,37,38,39]. The difference could be partially explained by the cost of HPV vaccination, as it was free in the United States while it was charged at market price with a slight discount in this study. It was likely that COVID-19 had a negative impact on HPV vaccination uptake in this study, as one third of unvaccinated participants mentioned concerns about COVID-19 transmission as a reason of not taking up HPV vaccination at Month 12.

The findings also had some implications to health service and health care policy. Our results supported that HPV vaccination was accepted by local MSM at the market rate. Providing subsidized or free HPV vaccination would further increase the uptake rate. MSM in Australia and the United Kingdom responded well to pilot programs providing free HPV vaccination [23,24,25]. Hong Kong should consider a similar pilot program for MSM, which would largely increase HPV vaccination coverage in this group and reduce HPV-related disease burdens.

The planning of future programs can be facilitated by the experiences of the present study. We found that those with history of HPV infection and/or genital warts were more responsive to the health promotion as compared to MSM without such history. It is possible that MSM with such history would perceive a stronger need to protect themselves by taking up HPV vaccination. Future studies are needed to explore whether different strategies should be applied for MSM with and without prior experience of HPV infection/genital warts. We also found that the baseline measurement of perceived susceptibility, perceived severity, perceived benefit, perceived self-efficacy, and behavioral intention related to HPV vaccination significantly predicted HPV vaccination uptake during the project period. 

Since our health promotion was standard and one-off, such findings suggested that some follow-ups should be implemented to modify these perceptions. Meta-analysis suggested that interventions tailored to one’s stage of change (SOC) were more effective than non-stage-tailored, especially among less motivated individuals [40]. Studies also showed that people might move forward to a higher SOC, go back to a lower SOC, or stay in the same SOC after exposing to the health promotion [41]. Therefore, future programs should consider stage-tailored health promotion with multiple sessions, which might be helpful to strengthen the perceived threat of HPV and perceived benefit of HPV vaccination. 

Moreover, asking people to provide where, when, and how they want to perform a behavior could increase the perceived self-efficacy [42]. The results of the process evaluation also provide insights for improving health promotion and procedures to receive HPV vaccination in future program. Future programs should consider crowdsourcing, which allows experts and target audience to share solutions together to increase the attractiveness of the contents [43]. Half of the MSM were not satisfied about HPV vaccination service providers’ acceptance of their subculture. Interventions targeting service providers to enhance their knowledge about MSM’s subculture is needed, and there were effective interventions in the literature [44]. Since the amount of HPV vaccination service providers would be relatively small, training to improve the situation should be feasible.

This study had the strengths of a relatively low dropout rate, validated primary outcome, and good process evaluation. However, it had several limitations. First, there was no control or comparison group. The aim of this study was to evaluate the effectiveness of the service model in real-world setting. Second, a selection bias existed, as we were not able to collect information from MSM who refused to join the project. They might have different characteristics comparing to participants. Third, attrition bias existed. The dropouts were more likely to be HIV positive or with unknown sero-status and had anal intercourse with NRP and male sex workers at the baseline. However, the bias should be limited as these baseline characteristics did not significantly predict HPV vaccination uptake during the project period. 

Fourth, we did not ask for details about how COVID-19 influenced the participants’ HPV vaccination. However, a previous study exploring the difficulties to access HIV-related services among MSM in Hong Kong during the same period provided some insights. Worrying about being exposed to a potentially COVID-19 infectious environment, experiencing disruptions in work due to COVID-19 and its control measures, and reduced connection to the MSM community during the pandemic were associated with increased difficulties in accessing HIV-related services in general [45]. Some of these factors might have negatively affected HPV vaccination among our participants. Finally, the study was based on a convenient sample of MSM, cautions should be taken when generalizing the results to MSM in Hong Kong.

## 5. Conclusions

The CBO-private clinic service model was helpful in increasing HPV vaccination uptake among MSM in Hong Kong. A larger-scale pilot program should be considered based on the experience of this project.

## Figures and Tables

**Table 1:** Baseline characteristics of the participants.

	All Participants (*n* = 350)	Being Followed Up at Month 12 (*n* = 274)	Dropouts (*n* = 76)	*p* Values
	*n* (%)	*n* (%)	*n* (%)	
**Socio-demographics**				
Age group (years)				
18–24	48 (13.7)	34 (12.4)	14 (18.4)	0.34
25–30	128 (36.6)	106 (38.7)	22 (28.9)	
31–40	133 (38.0)	103 (37.6)	30 (39.5)	
>40	41 (11.7)	31 (11.3)	10 (13.2)	
Relationship status				
Currently single	265 (75.7)	210 (76.6)	55 (72.4)	0.44
Married or cohabited with a man	85 (24.3)	64 (23.4)	21 (27.6)	
Highest education level attained				
Secondary or below	47 (13.4)	37 (13.5)	10 (13.2)	0.94
Tertiary of above	303 (86.6)	237 (86.5)	66 (86.8)	
Employment status				
Full-time	290 (82.9)	229 (83.6)	61 (80.3)	0.50
Part-time/unemployed/retired/students	60 (17.1)	45 (16.4)	15 (19.7)	
Monthly personal income, HK$ (US$)				
<10,000 (1282)	37 (10.6)	27 (9.9)	10 (13.2)	0.28
10,000–19,999 (1282–2564)	106 (30.3)	87 (31.8)	19 (25.0)	
20,000–39,999 (2565–5128)	134 (38.3)	107 (39.1)	27 (35.5)	
40,000 (5129)	67 (19.1)	50 (18.2)	17 (22.4)	
Refuse to disclose	6 (1.7)	3 (1.1)	3 (3.9)	
Sexual orientation				
Homosexual	319 (91.1)	252 (92.0)	67 (88.2)	0.30
Bisexual	31 (8.9)	22 (8.0)	9 (11.8)	
**Lifestyles**				
Smoking in lifetime				
No	257 (73.4)	203 (74.1)	54 (71.1)	0.60
Yes	93 (26.6)	71 (25.9)	22 (28.9)	
Drinking in the past year				
No	86 (24.6)	64 (23.4)	22 (28.9)	0.32
Yes	264 (75.4)	210 (76.6)	54 (71.1)	
**History of HIV and other STIs and service utilization**				
Self-reported HIV sero-status				
Negative	311 (88.9)	251 (91.6)	60 (78.9)	0.02
Positive	6 (1.7)	4 (1.5)	2 (2.6)	
Refuse to disclose	11 (3.1)	7 (2.6)	4 (5.3)	
Had never tested for HIV antibody	22 (6.3)	12 (4.4)	10 (13.2)	
History of HPV infection and/or genital warts (Yes)	31 (8.9)	27 (9.9)	4 (5.3)	0.21
History of other STIs (Yes)	59 (16.9)	47 (17.2)	12 (15.8)	0.78
Utilization of other HIV/STI prevention services (e.g., receiving free condoms, peer education and pamphlets, and attending seminars) (Yes)	81 (23.1)	69 (25.2)	12 (15.8)	0.09
**Sexual behaviors in the past six months**				
Anal intercourse with regular male sex partners (Yes)	293 (83.7)	230 (83.9)	63 (82.9)	0.83
Anal intercourse with non-regular male sex partners (Yes)	170 (48.6)	124 (45.3)	46 (60.5)	0.02
Anal intercourse with male sex workers (Yes)	9 (2.6)	4 (1.5)	5 (6.6)	0.01
Condomless anal intercourse with men (Yes)	176 (50.3)	142 (51.8)	34 (44.7)	0.27
Multiple male sex partnerships (Yes)	191 (54.6)	143 (52.2)	48 (63.2)	0.09
Sexual intercourse with female sex partners (Yes)	6 (1.7)	3 (1.1)	3 (3.9)	0.09
Condomless sex with female sex partners (Yes)	3 (0.9)	2 (0.7)	1 (1.3)	0.62
Sexualized drug use (use of psychoactive substances before or during sexual intercourse) (Yes)	20 (5.7)	17 (6.2)	3 (3.9)	0.45
**Knowledge related to HPV or HPV vaccination**				
Both males and females could be affected by HPV				
Yes ^a^	335 (95.7)	264 (96.4)	71 (93.4)	0.11
No	5 (1.4)	2 (0.7)	3 (3.9)	
Do not know	10 (2.9)	8 (2.9)	2 (2.6)	
HPV infection could cause STI				
Yes ^a^	317 (90.6)	248 (90.5)	69 (90.8)	0.79
No	8 (2.3)	7 (2.6)	1 (1.3)	
Do not know	25 (7.1)	19 (6.9)	6 (7.9)	
HPV infection could cause cancers among males				
Yes ^a^	258 (73.7)	200 (73.0)	58 (76.3)	0.54
No	27 (7.7)	20 (7.3)	7 (9.2)	
Do not know	65 (18.6)	54 (19.7)	11 (14.5)	
HPV could be totally cured by available treatment				
Yes	62 (17.7)	48 (17.5)	14 (18.4)	0.93
No ^a^	171 (48.9)	133 (48.5)	38 (50.0)	
Do not know	117 (33.4)	93 (33.9)	24 (31.6)	
Availability of effective HPV vaccination for males in Hong Kong				
Yes ^a^	288 (82.3)	229 (83.6)	59 (77.6)	0.12
No	8 (2.3)	4 (1.5)	4 (5.3)	
Do not know	54 (15.4)	41 (15.0)	13 (17.1)	
Number of shots required to prevent HPV infection in males				
3 ^a^	176 (50.3)	135 (49.3)	41 (53.9)	
Other answers/Do not know	174 (49.7)	139 (50.7)	35 (46.1)	
Number of correct responses, mean (SD)	4.4 (1.3)	4.4 (1.3)	4.4 (1.2)	0.91
**Perceptions related to HPV or HPV vaccination based on the HBM**				
Perceived susceptibility to HPV (high/very high)				
Perceived risk of contracting HPV in lifetime	89 (25.4)	65 (23.7)	24 (31.6)	0.16
Perceived risk of contracting genital warts in lifetime	72 (20.6)	50 (18.2)	22 (28.9)	0.04
Perceived risk of having penile/anal cancers in lifetime	42 (12.0)	32 (11.7)	10 (13.2)	0.73
Perceived Susceptibility Scale ^b^, mean (SD)	8.2 (2.6)	8.1 (2.6)	8.8 (2.7)	0.35
Perceived severity of HPV-related diseases (agree/strongly agree)				
HPV infection would increase risk of HIV acquisition	106 (30.3)	81 (29.6)	25 (32.9)	0.58
HPV infection would cause penile or anal cancers	127 (36.3)	98 (35.8)	29 (38.2)	0.70
Genital warts would have severe harms on your health	182 (52.0)	140 (51.1)	42 (55.3)	0.52
Penile or anal cancers would have severe harms on your health	263 (75.1)	207 (75.5)	56 (73.7)	0.74
Perceived Severity Scale ^c^, mean (SD)	13.4 (3.1)	13.3 (3.2)	13.8 (2.7)	0.43
Perceived benefits of HPV vaccination (agree/strongly agree)				
HPV vaccination is highly effective in preventing HPV infection	273 (78.0)	210 (76.6)	63 (82.9)	0.24
HPV vaccination is highly effective in preventing genital warts	240 (68.6)	181 (66.1)	59 (77.6)	0.054
HPV vaccination is highly effective in preventing penile/anal cancers	214 (61.1)	166 (60.6)	48 (63.2)	0.68
HPV vaccination can protect you for a long time	193 (55.1)	149 (54.4)	44 (57.9)	0.59
Perceived Benefit Scale ^d^, mean (SD)	15.3 (2.5)	15.2 (2.6)	15.6 (2.1)	0.20
Perceived barriers of receiving HPV vaccination (agree/strongly agree)				
It is not worthy speeding HK$6000–7000 (US$774–903) to receive HPV vaccination	107 (30.6)	88 (32.1)	19 (25.0)	0.23
You would have severe side-effects after receiving HPV vaccination	37 (10.6)	30 (10.9)	7 (9.2)	0.66
Others would think you are having high-risk behaviors if you receive HPV vaccination	45 (12.9)	37 (13.5)	8 (10.5)	0.49
You would be stigmatized when you receive HPV vaccination	34 (9.7)	26 (9.5)	8 (10.5)	0.79
If you already infected with HPV, HPV vaccination could not protect you	111 (31.7)	88 (32.1)	23 (30.3)	0.76
Perceived Barrier Scale ^e^, mean (SD)	12.4 (3.1)	12.4 (3.1)	12.2 (3.5)	0.76
Perceived cue to action related to HPV vaccination (agree/strongly agree)				
Mass media suggest males to receive HPV vaccination	62 (17.7)	46 (16.8)	16 (21.1)	0.39
People who are important to you would suggest you to receive HPV vaccination	83 (23.7)	66 (24.1)	17 (22.4)	0.76
Cue to Action Scale ^f^, mean (SD)	4.9 (2.0)	4.8 (2.0)	5.2 (2.0)	0.14
Perceived self-efficacy related to HPV vaccination (agree/strongly agree)				
You are confident to receive HPV vaccination in the next year if you want	160 (45.7)	129 (47.1)	31 (40.8)	0.33
Receiving HPV vaccination in the next year is easy for you if you want	182 (52.0)	148 (54.0)	34 (44.7)	0.15
Perceived Self-efficacy Scale ^g^, mean (SD)	6.8 (2.0)	6.9 (2.0)	6.7 (2.0)	0.37
**Behavioral intention to take up HPV vaccination (likely/very likely)**				
Likelihood of taking up three required doses of HPV vaccines in the next year	64 (18.3)	52 (19.0)	12 (15.8)	0.53

^a^ Correct response. ^b^ Perceived Susceptibility Scale: three items, Cronbach’s alpha: 0.85, one factor was identified by exploratory factor analysis, explaining for 77.2% of total variances. ^c^ Perceived Severity Scale: four items, Cronbach’s alpha: 0.72, one factor was identified by exploratory factor analysis, explaining for 54.9% of total variances. ^d^ Perceived Benefit Scale: four items, Cronbach’s alpha: 0.79, one factor was identified by exploratory factor analysis, explaining for 62.4% of total variances. ^e^ Perceived Barrier Scale: five items, Cronbach’s alpha: 0.62, one factor was identified by exploratory factor analysis, explaining for 57.9% of total variances. ^f^ Cue to Action Scale: two items, Cronbach’s alpha: 0.61, one factor was identified by exploratory factor analysis, explaining for 56.2% of total variances. ^g^ Perceived Benefit Scale: two items, Cronbach’s alpha: 0.79, one factor was identified by exploratory factor analysis, explaining for 82.5% of total variances. HK: Hong Kong; HIV: human immunodeficiency virus; STI: sexually transmitted infection; and HPV: human papillomavirus.

**Table 2:** Baseline background characteristics predicting human papillomavirus vaccination uptake (among participants who completed Month 12 follow-up, *n* = 274).

	OR (95%CI)	*p* Values
**Socio-demographics**		
Age group (years)		
18–24	1.0	
25–30	0.80 (0.32, 2.03)	0.64
31–40	0.43 (0.16, 1.16)	0.10
>40	0.63 (0.18, 2.17)	0.46
Relationship status		
Currently single	1.0	
Married or cohabited with a man	0.77 (0.35, 1.68)	0.51
Highest education level attained		
Secondary or below	1.0	
Tertiary of above	1.78 (0.60, 5.29)	0.30
Employment status		
Full-time	1.0	
Part-time/unemployed/retired/students	0.57 (0.21, 1.54)	0.27
Monthly personal income, HK$ (US$)		
<10,000 (1282)	1.0	
10,000–19,999 (1282–2564)	1.53 (0.41, 5.80)	0.53
20,000–39,999 (2565–5128)	1.73 (0.47, 6.33)	0.41
40,000 (5129)	2.00 (0.50, 8.00)	0.33
Refuse to disclose	N.A.	N.A.
Sexual orientation		
Homosexual	1.0	
Bisexual	1.11 (0.36, 3.45)	0.86
**Lifestyles**		
Smoking in lifetime		
No	1.0	
Yes	0.88 (0.42, 1.84)	0.74
Drinking in the past year		
No	1.0	
Yes	0.96 (0.46, 2.03)	0.92
**History of HIV and other STIs and service utilization**		
Self-reported HIV sero-status		
Negative	1.0	
Positive	5.28 (0.72, 38.55)	0.10
Refuse to disclose	3.96 (0.85, 18.36)	0.08
Had never tested for HIV antibody	0.49 (0.06, 3.82)	0.49
History of HPV infection and/or genital warts		
No	1.0	
Yes	6.02 (2.60, 13.94)	<0.001
History of other STIs (Yes)		
No	1.0	
Yes	1.02 (0.44, 2.36)	0.96
Utilization of other HIV/STI prevention services (e.g., receiving free condoms, peer education and pamphlets, and attending seminars)		
No	1.0	
Yes	1.98 (1.01, 3.89)	0.046
**Sexual behaviors in the past six months**		
Anal intercourse with regular male sex partners		
No	1.0	
Yes	1.08 (0.45, 2.60)	0.87
Anal intercourse with non-regular male sex partners		
No	1.0	
Yes	1.55 (0.82, 2.93)	0.18
Anal intercourse with male sex workers		
No	1.0	
Yes	1.67 (0.17, 16.39)	0.66
Condomless anal intercourse with men		
No	1.0	
Yes	1.26 (0.66, 2.38)	0.49
Multiple male sex partnerships		
No	1.0	
Yes	1.91 (0.99, 3.69)	0.06
Sexual intercourse with female sex partners		
No	1.0	
Yes	N.A.	N.A.
Condomless sex with female sex partners		
No	1.0	
Yes	N.A.	N.A.
Sexualized drug use (use of psychoactive substances before or during sexual intercourse)		
No	1.0	
Yes	1.58 (0.49, 5.07)	0.45

OR: crude odds ratios; CI: confidence interval; HK: Hong Kong; HIV: human immunodeficiency virus; STI: sexually transmitted infection; HPV: human papillomavirus; and N.A.: not applicable.

**Table 3:** Factors predicting human papillomavirus vaccination uptake (among participants who completed Month 12 follow-up, *n* = 274).

	OR (95%CI)	*p* Values	AOR (95%CI)	*p* Values
**Knowledge related to HPV or HPV vaccination**				
Number of correct responses	1.29 (0.97, 1.70)	0.08	1.15 (0.86, 1.54)	0.35
**Perceptions related to HPV or HPV vaccination based on the HBM**				
Perceived Susceptibility Scale	1.31 (1.15, 1.49)	<0.001	1.25 (1.09, 1.43)	0.002
Perceived Severity Scale	1.19 (1.06, 1.33)	0.003	1.21 (1.07, 1.37)	0.003
Perceived Benefit Scale	1.19 (1.05, 1.36)	0.009	1.16 (1.01, 1.33)	0.03
Perceived Barrier Scale	0.88 (0.79, 0.98)	0.02	0.90 (0.80, 1.01)	0.07
Cue to Action Scale	1.16 (0.99, 1.36)	0.08	1.10 (0.93, 1.30)	0.26
Perceived Self-efficacy Scale	1.42 (1.19, 1.71)	<0.001	1.37 (1.14, 1.65)	0.001
**Behavioral intention to take up HPV vaccination**				
Likelihood of taking up three required doses of HPV vaccines in the next year				
Very unlikely/unlikely/neutral	1.0		1.0	
Likely/very likely	8.86 (4.38, 17.95)	<0.001	6.99 (3.34, 14.60)	<0.001

HPV: human papillomavirus; HBM: health belief model; OR: crude odds ratios; CI: confidence interval; and AOR: adjusted odds ratios, odds ratios adjusted for significant baseline background characteristics (i.e., history of HPV infection and/or genital warts, and utilization of other HIV/STI prevention services).

**Table 4:** Process evaluation of the health promotion and the procedures to receive human papillomavirus vaccination at private clinics.

	*n*	%
Process evaluation of the health promotion (among participants who completed the Month 12 follow-up evaluation, *n* = 274) (agree/strongly agree)		
The content of the health promotion is clear	198	72.3
The content of the health promotion is attractive	106	38.7
The health promotion has increased their understanding on benefit of HPV vaccination	201	73.4
The health promotion has reduced their barriers to take up HPV vaccination	126	46.0
The health promotion has increased their confidence to take up HPV vaccination	154	56.2
The health promotion has increased their willingness to take up HPV vaccination	111	40.5
Satisfaction with the following procedures to receive HPV vaccination at private clinics (among participants who had completed HPV vaccination during the follow-up period, *n* = 46) (satisfied/very satisfied)		
Convenience of the location of clinic	41	89.1
Convenience of the arranged time slot for vaccination	44	95.7
Waiting time between making appointment and receiving the first dose of HPV vaccination	39	84.8
Level of privacy	32	69.6
Private clinic staff’s acceptance of MSM’s subculture	23	50.0
Explanation made by the staff of the private clinic	34	73.9
Level of professionalism of the staff of the private clinic	35	76.1

HPV: human papillomavirus; and MSM: gay, bisexual, other men who have sex with men.

## Data Availability

The data presented in this study are available from the corresponding authors upon request. The data are not publicly available as they contain sensitive personal behaviors.
